# *Medicago sativa* L. Plant Response against Possible Eustressors (Fe, Ag, Cu)-TiO_2_: Evaluation of Physiological Parameters, Total Phenol Content, and Flavonoid Quantification

**DOI:** 10.3390/plants12030659

**Published:** 2023-02-02

**Authors:** Luis Páramo, Ana Angélica Feregrino-Pérez, Marina Vega-González, Luis Escobar-Alarcón, Karen Esquivel

**Affiliations:** 1 División de Investigación y Posgrado, Facultad de Ingeniería, Universidad Autónoma de Querétaro, Cerro de las Campanas, Santiago de Querétaro 76010, Mexico; 2 Centro de Geociencias, Universidad Nacional Autónoma de México, Campus Juriquilla. Blvd. Juriquilla, 3001, Santiago de Querétaro 76230, Mexico; 3 Departamento de Física, ININ, Carr. México-Toluca, La Marquesa, Ocoyoacac 52750, Mexico

**Keywords:** abiotic stress, *Medicago sativa* L., physiological changes, secondary metabolites, titanium dioxide

## Abstract

The present study analyzed *Medicago sativa* L. crops irrigated by TiO_2_ in the anatase phase and TiO_2_ doped with Ag, Fe, and Cu ions at 0.1%w synthesized using the sol–gel method (SG) and the sol–gel method coupled with microwave (Mw-SG). The materials were added to the irrigation water at different concentrations (50, 100, and 500 ppm). Stress induction by nanomaterials was observed by measuring stem morphology, chlorophyll index, total phenols and flavonoids, and antioxidant activity through the DPPH (2,2-diphenyl-1-picrylhydrazy) radical inhibition assay. The nanomaterial treatments caused statistically significant reductions in parameters such as stem length, leaf size, and chlorophyll index and increases in total phenol content and DPPH inhibition percentage. However, the observed effects did not show clear evidence regarding the type of nanomaterial used, its synthesis methodology, or a concentration-dependent response. By generally grouping the results obtained to the type of dopant used and the synthesis method, the relationship between them was determined employing a two-way ANOVA. It was observed that the dopant factors, synthesis, and interaction were relevant for most treatments. Additionally, the addition of microwaves in the synthesis method resulted in the largest number of treatments with a significant increase in the total content of phenols and the % inhibition compared to the traditional sol–gel synthesis. In contrast, parameters such as stem size and chlorophyll index were affected under different treatments from both synthesis methods.

## 1. Introduction

When interacting with plants, nanomaterials (NMs), such as metals, metal oxides, polymers, or carbon structures, can generate different degrees of stress, which can lead to eustressic or distressic effects [1]. Within the area of stress induction by nanomaterials, the effects caused can be exploited for the enhancement of metabolic pathways that reflect on plant characteristics such as a larger size, improved nutritional content, and enhanced defense against biotic and abiotic stresses, among others [2,3].

Nanomaterials possess a variety of physicochemical features that lead the pathway to stress in plants and, therefore, to the positive or negative effects abovementioned [1]. Physicochemical characteristics such as size, surface area, dose, concentration, charge, and crystalline structure influence the generation of positive or negative effects on plant physiology and metabolic activity; however, the stress response will also be related to plant characteristics, such as age, tissue characteristics, route of exposure, molecules present in the media and additional stresses [1,4,5]. Because NPs’ characteristics influence plants’ responses, studies investigating the varied physicochemical characteristics and their influence on plants are adequate to recognize and relate how these characteristics relate to the observed effects in such a way that NPs features can be adjusted to obtain better results in plant development [6].

The alteration in secondary metabolite content is one of the main areas of NPs used in agriculture; secondary metabolites are molecules associated with defense mechanisms in plants and increase their production when they are affected by biotic and abiotic stress [7,8]. Nanomaterials, through multiple mechanisms such as reactive oxygen species (ROS) generation, have proven to be sources of stress induction [9], which under appropriate conditions, can cause an increase in the content of secondary metabolites without having more significant effects on other characteristics of the plants, leading to the generation of value-added products [10].

Nanomaterials such as metal oxides have proven to be suitable for producing plants with better characteristics [2]. Among these materials, TiO_2_ has varied effects on various crops, offering positive aspects on plant development and increasing characteristics such as size, mass, and secondary metabolite content [11,12]. The benefits of using TiO_2_ in plants are reflected in multiple investigations proving to be efficient products for developing crops with higher nutrient content and increased valuable compounds [13,14,15]. Plants such as *Stevia rebaudiana* Bertoni, *Mentha piperita* L., *Saponaria officinalis* L., and *Triticum aestivum* L. have improved features when interacting with TiO_2_ NPs, increasing growth, germination, soluble sugar and proteins content, photosynthetic pigments, and phenolic contents, even if the plant was submitted to additional stresses such as salinity [16,17,18,19]. Although, some results indicate that TiO_2_ can lead to adverse effects when increasing the concentrations, affecting germination and yield [19].

To our knowledge, the effect of TiO_2_ on alfalfa and how different physicochemical characteristics alter its properties and characteristics has not been investigated. This work aimed to evaluate possible eustressic effects caused by TiO_2_ materials synthesized by two different pathways and with different doping characteristics (Cu, Fe, Ag), showing how they relate to alfalfa morphology, chlorophyll index, total phenol, flavonoid content, antioxidant activity, and how factors such as synthesis and dopant relate to the effects observed.

## 2. Results

### 2.1. Physicochemical Characterization of TiO_2_ Materials

**Figure 1:**
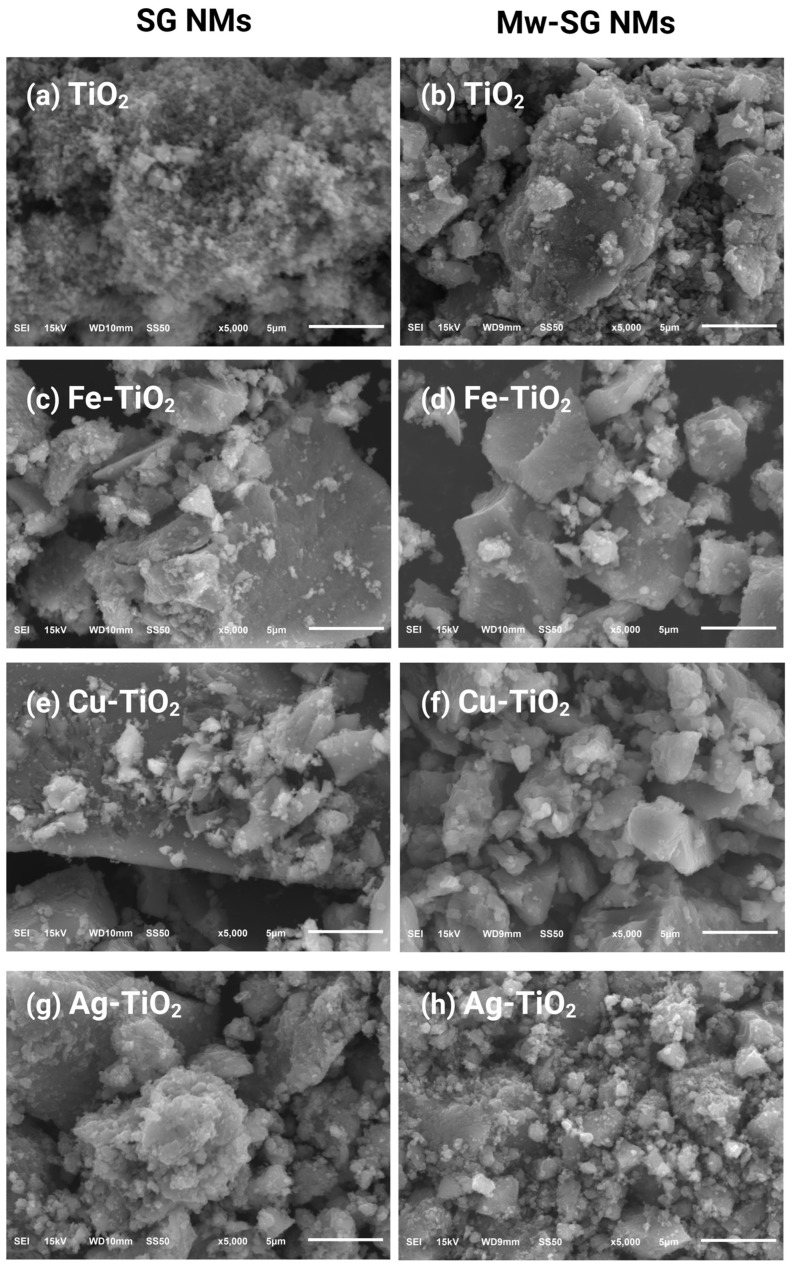
SEM images (5000×) of the SG- and Mw-SG-synthesized materials, (**a**,**b**) TiO_2_, (**c**,**d**) Fe-TiO_2_, (**e**,**f**) Cu-TiO_2_, (**g**,**h**) Ag-TiO_2_.

Figure 1 shows a structural comparison between the materials synthesized by SG and Mw-SG. The synthesized materials by Mw-SG corresponding to Figure 1b,d,f,h show aggregates of lesser size than the pure sol–gel method (Figure 1a,c,e,g). The internal heat generated through this process helps form NMs with high crystallinity and small and uniform size.

**Figure 2:**
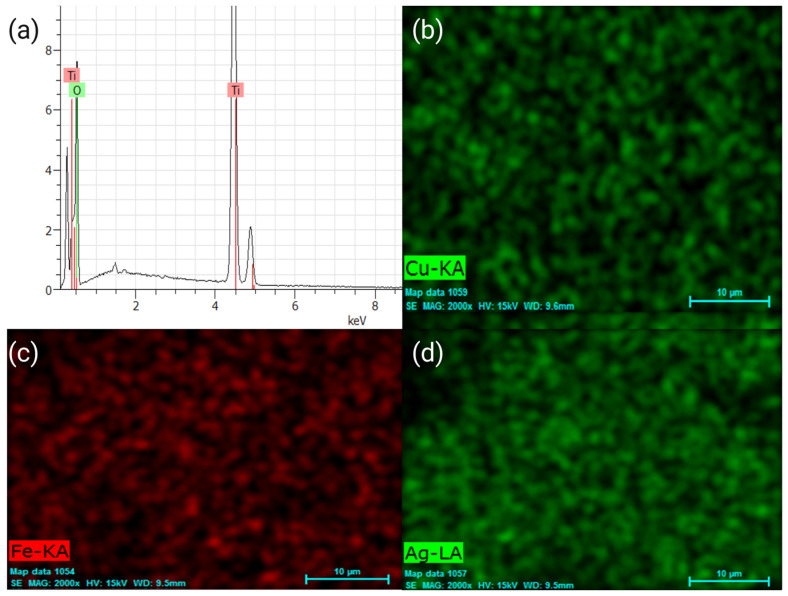
Elemental analysis of the (**a**) SG-TiO_2_ and elemental mappings of (**b**) Cu, (**c**) Fe, and (**d**) Ag-doped TiO_2_.

EDS shows the elemental composition of the M-TiO_2_, M = Ag, Fe, and Cu NPs, synthesized using the sol–gel method, where the presence of the Ti and O elements can be seen in Figure 2a for the undoped TiO_2_. In Figure 2b–d, the elemental mapping for the doped materials is observed, where the presence of the elements Cu (Figure 2b), Fe (Figure 2c), and Ag (Figure 2d) are observed. Identical results were obtained for the Mw-SG synthesis method.

**Figure 3:**
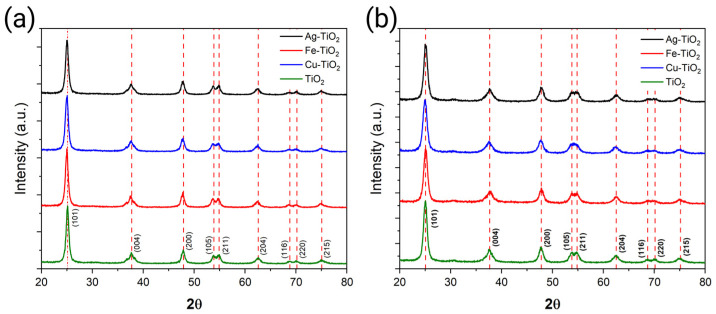
X-ray diffraction patterns of (**a**) SG-synthesized and (**b**) Mw-SG-synthesized materials.

X-ray diffraction patterns for the SG- and Mw-SG-synthesized materials are shown in Figure 3a,b. The diffraction peaks at 2θ angles of 25.1°, 37.7°, 47.8°, 53.6°, 54.8°, 62.5°, 68.7°, 70.1°, and 75.1° are related to the anatase phase of TiO_2_, no presence of rutile or brookite phase was observed based on the diffraction signals. No diffraction peaks related to the dopants due to their low concentration were also observed.

The crystallite size for both synthesis methods is compiled in Table 1, where the Scherrer equation gave an average of 9 nm crystallite size for the SG NMs and 7.49 nm for the Mw-SG. On the other hand, the Williamson–Hall equation shows an average crystallite size of 17.2 nm for the SG NMs and 12.12 nm for the Mw-SG NMs.

**Figure 4:**
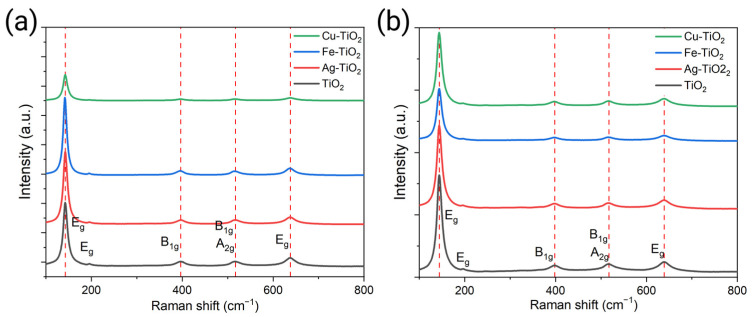
Raman spectra of (**a**) SG-synthesized and (**b**) Mw-SG-synthesized materials.

The crystal phase was also confirmed by Raman spectroscopy. The spectra of sol–gel NMs are shown in Figure 4a. Mw-SG- (Figure 4b) and SG-synthesized materials show four signals at 142.7 (E_g_), 396.8 (B_1g_), 517 (B_1g_/A_1g_), and 637.7 (E_g_) cm^−1^, which are indicative of the presence of the anatase crystalline phase, without the presence of the brookite and rutile phases of the TiO_2_. No band shifting or new signals are observed due to the low concentration of dopants.

### 2.2. Morphological Data

**Figure 5:**
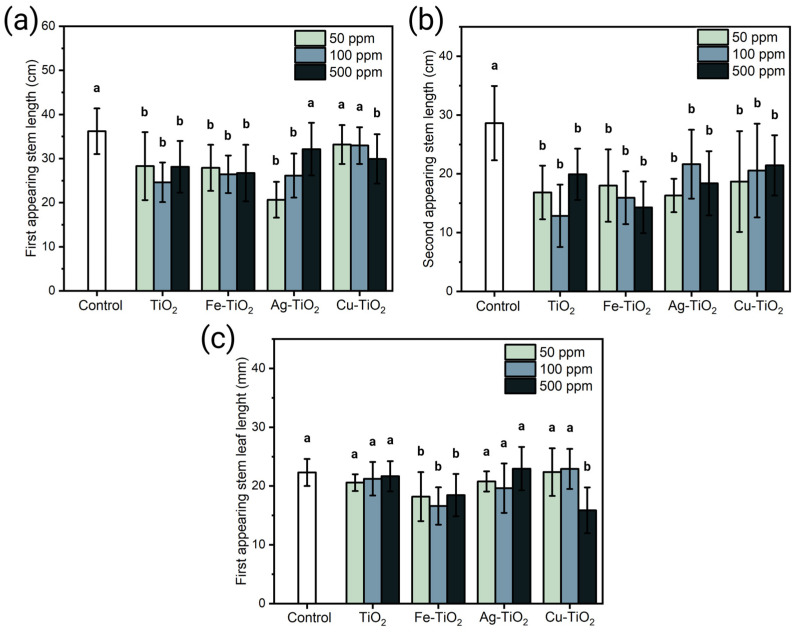
(**a**) Central stem length and (**b**) secondary stem length (**c**) leaf length of plants treated with SG-synthesized NMs for 80 days. Comparison between means was analyzed using a Dunnett assay with a *p* ≤ 0.05; means sharing a letter are considered not statistically different.

The stem length of the alfalfa crops was measured in the first and second appearing stems. Figure 5a contains the measurements obtained from the length (cm) of the central stem for plants treated with SG NMs. In general, it is observed that the treatments meant a minimum reduction of 10% and a maximum of 30% compared to the control. The pairwise analysis to control (Dunnett) indicates that the reductions observed for the three concentrations of TiO_2_ and Fe-TiO_2_ are significantly statistical; however, in the Ag-TiO_2_ materials, the concentration of 500 ppm, as well as 50 and 100 ppm for Cu-TiO_2_, do not indicate the significant difference with the control.

The measurements obtained from the second appearing stem (Figure 5b) also showed a size reduction. In this case, all the types of NMs in their three concentrations meant a statistically significant reduction in the average size of the second stem, reaching a general decrease of approximately 35%. On the other hand, the leaf length (Figure 5c) was also affected by the different NPs treatments, where materials such as TiO_2_ and Ag-TiO_2_ did not mean a statistically significant reduction, compared to Fe-TiO_2_ were at its three concentrations caused an average decrease of 25%. On the other hand, the maximum reduction was obtained at 500 ppm with Cu-TiO_2_, whose first two concentrations did not generate a significant change.

**Figure 6:**
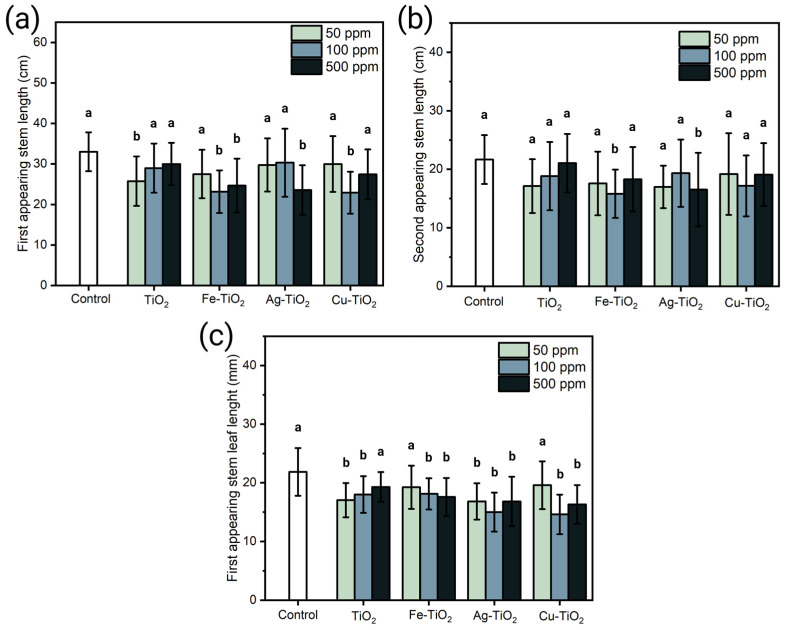
(**a**) Central stem length and (**b**) secondary stem length (**c**) leaf length of plants treated with Mw-SG-synthesized NMs for 80 days. Comparison between means was analyzed using a Dunnett assay with a *p* ≤ 0.05; means sharing a letter are considered not statistically different.

In the same way as the plants treated with NMs SG, the Mw-SG materials caused a reduction in parameters, such as the length of the first stem, to appear, as shown in Figure 6a. In general, the treatments of the different types of NMs caused an average reduction of 12% with a maximum reduction of 28%. A pairwise comparison to the control reveals that the materials used exerted a statistically significant abatement at different concentrations, such as 50 ppm for TiO_2_, 100 ppm for Fe-TiO_2_ and Cu-TiO_2_, and 500 ppm for Fe-TiO_2_. TiO_2_ and Ag-TiO_2_.

Data on the length of the second stem to appear (Figure 6b) indicated that materials such as TiO_2_ and Cu-TiO_2_ caused a non-statistically significant reduction in its three concentrations. In contrast, Fe-TiO_2_ and Ag-TiO_2_ only caused a statistically significant reduction of approximately 30% in 100 and 500 ppm concentrations, respectively. Finally, the effect observed in leaf length (Figure 6c) indicates a general lowering, which is significant for the Ag-TiO_2_ treatments in all concentrations. At the same time, TiO_2_ exerted a significant impact at 50 ppm and 100 ppm. On the other hand, Fe-TiO_2_ and Cu-TiO_2_ exerted a statistically significant reduction at 100 and 500 ppm.

### 2.3. Total Phenol and Flavonoid Quantification

To evaluate the effect of NMs on crop growth, the secondary metabolite content in leaves, stems, and roots was carried out and presented in Table 2. The effect of the MNs on the gallic acid content in the three organs analyzed was treated by 1-way ANOVA and treated with a Dunnett test, comparing the treatment with the control. The leaves of the plants treated with NMs SG generally show that the plants treated with NMs have a higher gallic acid content. However, statistical analysis reveals that none of the 50 ppm treatments caused a significant effect at the lowest concentration, with TiO_2_ being the only material to generate a relevant increase of approximately 70 and 36% for the 100 and 500 ppm concentrations, respectively.

On the other hand, the gallic acid content suffered an increase for the plants treated with NMs; however, at 50 ppm, none of the treatments were considered statistically significant. At 100 ppm, materials such as TiO_2_, Ag-TiO_2_, and Cu-TiO_2_ caused an average increase of 40%, while at 500 ppm, only TiO_2_ caused a significant increase of 50%. Root analysis shows a significant 70% increase at 50 ppm for TiO_2_. At 100 ppm, the TiO_2_, Ag-TiO_2_, and Cu-TiO_2_ materials reflected a substantial increase of more than 50%. Finally, at 500 ppm, TiO_2_ and Cu-TiO_2_ were the only statistically significant treatments, with an increase greater than 50%.

The content of total flavonoids measured, expressed as mg eq. rutin, showed atypical results where the content in mg was higher than the total phenolic content, which can be considered a misinterpretation as it is a subcategory of phenolic compounds. This higher response is associated with the standard, which may overestimate the quantified content. Despite this, statistical analysis revealed that these were not statistically significant despite showing an increase in total flavonoid content in most treatments compared to the control (Table S1).

The gallic acid content in the leaves of the plants treated with Mw-SG NMs (Table 3) suffered an increase where at 50 ppm, the TiO_2_, Ag-TiO_2_, and Fe-TiO_2_ treatments caused a statistically significant increase of more than 30%. On the other hand, at 100 ppm, all treatments caused a significant increase of at least 28%. Finally, at 500 ppm, the four NMs treatments resulted in a statistically significant increase to the control, increasing the gallic acid content between 27 and 51%. In the case of the stem, materials such as Fe-TiO_2_ and Cu-TiO_2_ did not cause a significant increase in gallic acid content. At 100 ppm, only the TiO_2_ and Fe-TiO_2_ treatments resulted in a statistically significant increase of at least 67%. Finally, at 500 ppm, only the TiO_2_ treatment was not statistically significant, whereas the other materials caused a rise of at least 40%. Finally, the observed effect of the treatments on the roots indicates that none of the treatments caused a statistically significant impact at 50 ppm. In comparison, at 100 and 500 ppm, only the Cu-TiO_2_ material caused a significant increase of approximately 70% for both concentrations.

For Mw-SG NMs, the flavonoid content suffered a general increase in all the treatments of up to 80%; however (Table S2), most treatments were not statistically different, maintaining their total content in mg higher than the total content of phenols. On roots, the content of total flavonoids was statistically different for the Cu-doped materials at 500 ppm, causing an increase of 41.99%.

### 2.4. Antioxidant Activity

The statistical analysis of the leaf parameters (Table 4) reveals that at a concentration of 50 and 100 ppm, the NM TiO_2_ was the only one that caused a significant increase of approximately 40 and 50%, respectively. At the same time, the other materials did not generate relevant values along the three connections. On the other hand, the % of IHB in the stem was affected by significant multiple treatments caused at 50 ppm by TiO_2_ and Ag-TiO_2_, displaying a minimum increase of 50%; at concentrations of 100 ppm, only the treatment with TiO_2_ resulted in a significant rise of 70%, while at 500 pm, said material caused a 57% increase. In contrast, the rest of the treatment was not statistically relevant. Finally, the effect observed in the roots indicates a modification in its antioxidant capacity. At 50 ppm, TiO_2_ and Fe-TiO_2_ generated an increase greater than 50%; at 100 ppm, the TiO_2_, Ag’TiO_2_, and Fe-TiO_2_ treatments caused a significant increase of more than 60%. Finally, at 50 ppm, all the materials significantly increased the antioxidant capacity of the roots by at least 65%.

For the SG-Mw-treated plant, IHB% was also increased (Table 5). However, no relation between an increasing NP concentration and an increase in radical inhibition was observed. The leaf analysis shows that at the three concentrations used, all the NMs were capable of increasing IHB% in a statistically significant matter, whereas at 50 and 100 ppm, the treatments such as Fe-TiO_2_ caused an increase of 30%, the highest increase being of 64% obtained at 50 ppm by TiO_2_. All the treatments in the stem proved significant again, achieving an increase of between 30 and 100%. Finally, the analysis in the roots shows that the NMs used at the three concentrations caused a significant increase of between 28 and 120%, except for the treatment at 50 ppm by Ag-TiO_2_.

### 2.5. Chlorophyll Index

**Figure 7:**
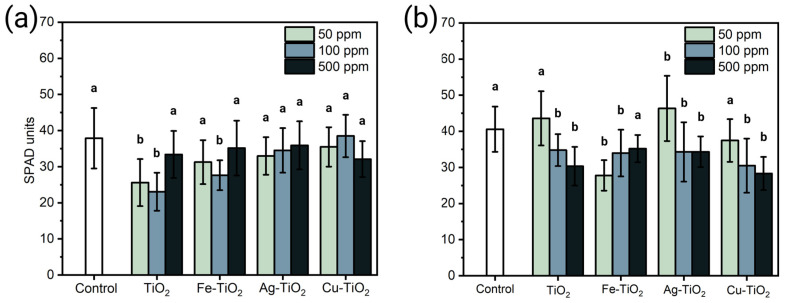
Chlorophyll index measure in SPAD units for alfalfa treated with (**a**) SG- and (**b**) Mw-SG-synthesized NMs. Comparison between means was analyzed using a Dunnett assay with a *p* ≤ 0.05; means sharing a letter are considered not statistically different.

In general, an apparent decrease in the chlorophyll index was observed (Figure 7a); however, the reduction was not statistically significant to the control. TiO_2_ material at a concentration of 50 and 100 ppm achieved a statistically significant decrease of approximately 30%. On the other hand, for the Fe-TiO_2_ material, only 100 ppm significantly reduced the chlorophyll index, achieving a 27% reduction.

The chlorophyll indices of the plants treated with the materials obtained by the Mw-SG synthesis method are shown in Figure 7b. It was observed that the chlorophyll content was significantly reduced at concentrations of 50 and 100 for the TiO_2_ and Cu-TiO_2_ treatments, generating a decrease more significant than 14%. The Fe-TiO_2_ material also caused a statistically significant decrease reflected at 50 and 100 ppm, causing a 15% decrease. Finally, the Ag-TiO_2_ material caused a behavior not previously observed where the chlorophyll index increased significantly to the control by 13%, while at 100 and 500 ppm, there was a similar reduction of 15%.

### 2.6. Two-Way ANOVA

Based on the previously shown results, the use of NMs meant a decrease in parameters such as stem size, leaf length, and chlorophyll index, in turn, an increase in the phenol content and the antioxidant activity present in the plant. The behavior of the observed results is not related to the rise in the concentration of NMs. Although some treatments show statistical significance compared to the control, a statistical comparison by pairs reveals that the effect observed between NMs treatment does not represent a significant difference. The results obtained in each concentration (50, 100, and 500 ppm) were considered mere replicates and grouped on their respective NMs. Under these considerations, two-way ANOVA analyzes were performed, normalizing data to control, taking as factors the dopant of each NM (undoped, Fe, Cu, and Ag), taking as repetitions the values collected from each concentration used, and the synthesis method (SG and MW). Keeping the control data makes it possible to corroborate whether the treatments have a significant effect, regardless of the concentration used. On the other hand, comparing the factors without using control data would indicate if the factors had a substantial difference between them, helping to observe if the presence of dopants influences the measured parameters compared to the undoped sample.

Two-way ANOVA analyses with and without control over the data obtained for the length of the first stem at appearance (Table 6) indicate that synthesis, doping, and the interaction of both factors are significant, suggesting a change in the nanoparticle because of the synthesis method and its respective doping. A Tukey pairwise test was performed to differentiate the effects of synthesis and dopant (Table S3). The results indicate that the four types of nanoparticles significantly reduced stem length for both synthesis methods. Nevertheless, there was a difference in the effect of doping-related growth inhibition where copper-SG caused a significant impact compared to TiO_2_. For the Mw-SG materials, a significant impact was observed with the Fe-TiO_2_, the other NMs having a non-significant response concerning TiO_2_.

The case of the second stem corresponding to the ANOVA analysis (Table S4), comparing the nanoparticles without control, shows that only the synthesis factor was significant. Furthermore, the Tukey analysis (Table S5) demonstrated the most significant influence on the length of the second stem for the SG materials. All the treatments were significantly statistically compared to Mw-SG, where only Ag-TiO_2_ and Cu-TiO_2_ modified the size. In turn, the null difference between the effect of the dopant on TiO_2_ for both synthesis methods.

The leaf phenol content showed that the analysis with control and without control significantly affected the synthesis and doping factors (Table 7). The comparative table with the Tukey analysis (Table S6) indicates a more significant effect for the Mw-SG materials, where all the treatments with dopants significantly increased the phenol content. However, there was no statistical difference between TiO_2_ and the rest of the dopants, which reveals the same effect on phenol content regardless of the used dopant. A lower influence was observed in the SG materials, where only the TiO_2_ material indicated a significant effect compared to the control. On the other hand, the comparative analysis showed a statistical difference between the Fe-TiO_2_ material and TiO_2_.

In the case of the phenolic content in the stem, there was no significant influence between the synthesis and dopant factors for the comparison without using the control (Table S7). The Tukey pairwise comparison (Table S8) shows no significant effect related to the SG NMs, while the Mw-SG materials, such as TiO_2_, Cu-TiO_2_, and Fe-TiO_2_, caused a significant change. The analysis of the phenolic content in roots was influenced by dopant and the interaction between factors (Table S9). In contrast, the pair analysis between materials significantly affected both parameters’ synthesis, dopant, and interaction (Table S10). The comparison by Tukey indicates that the NMs SG only have a significant effect due to TiO_2_. At the same time, for the Mw-SG, the Cu-TiO_2_ meant the greatest effect, which was statistically significant compared to TiO_2_.

The ANOVA analysis for the inhibition % in leaves shows that the doping factors, synthesis, and their interaction are significant; this is shown in the pairwise comparison (Table 8), where the most significant effect on this parameter is reflected in the Mw-SG materials being all the significant treatments, especially the Fe-TiO_2_. In the case of the SG materials, it can be seen how the TiO_2_ and Ag-TiO_2_ significantly changed concerning the control (Table S11).

These factors and their interaction remained statistically relevant for the other sections measured (stem and roots) (Tables S12 and S14), where it was also observed that the microwave synthesis method generated a more significant effect than the SG route.

The dopant and synthesis factors and their interaction remained significant for the effect observed in the chlorophyll index (Table 9). However, a clear difference concerning the general effect due to the synthesis method was not observed. In this case, it is shown in Table S15, the comparison by pairs, that for the SG materials, the most significant effect in the reduction in the chlorophyll index was by TiO_2_ and Fe-TiO_2_, while in the Mw-SG, it was by the materials Cu-TiO_2_ and Fe-TiO_2_.

## 3. Discussion

As mentioned above, NPs’ physicochemical characteristics guide the stress generation mechanisms, leading to plants’ biostimulation or inhibitory activity [1]. Through sol-gel synthesis and sol-gel microwave assisted, it was obtained TiO_2_ nanomaterials with different doping elements are present in their structure, as seen in EDS analysis. Furthermore, it was revealed that the followed route of synthesis as well as post-treatment resulted in structural modifications, as presented in Raman spectroscopy. A more detailed analysis of XRD patterns revealed that the addition of energy through microwaves leads to a smaller crystallite size [20] among all types of doped NPs, making their crystallite size, as seen through Williamson–Hall, more even as compared to sol–gel synthesis.

**Figure 8:**
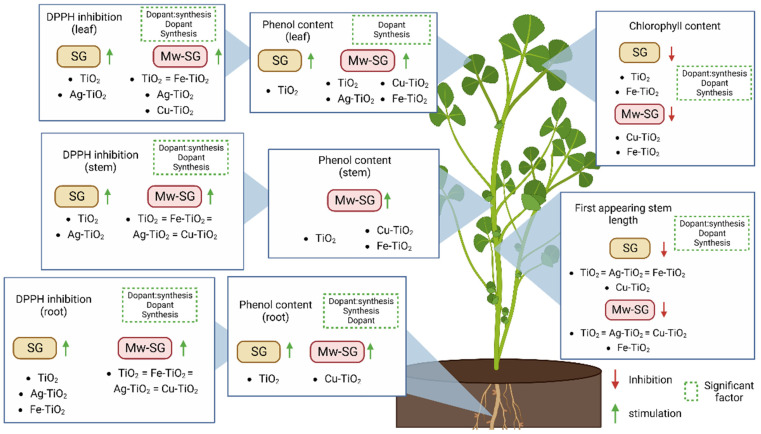
Résumé of the effects observed in alfalfa crops, NM treatments shown in the image caused a significant increase or reduction in the measured parameter compared to the control.

Dunnett’s analysis showed that specific treatments could significantly raise or lower the measured parameters; however, said treatments did not show a significant dependence on the concentration of nanomaterial supplied. In summary, two-way ANOVA analysis confirmed the influence of factors such as dopant, synthesis method, and their interaction, which were relevant in parameters such as chlorophyll index, stem size, and inhibition % in the three organs measured. Figure 8 summarizes the effects observed in the plant due to the treatments and indicates which of these seen by the pair test generated a significant impact concerning their normalized controls.

Parameters such as stem size indicated that both synthesis methods had an adverse effect under all applied treatments. On the other hand, the chlorophyll index was affected by individual treatments, which differed according to the synthesis method. The total content of phenols reflected a more significant difference depending on the properties of the NMs acquired by the synthesis method. Only the TiO_2_ SG caused a significant increase in this parameter in the three organs measured. By modifying the synthesis method, the NMs’ characteristics were changed, resulting in a more significant number of effective treatments, as in the content of phenols in leaves and stems. Said effect was also observed in the inhibition % of DPPH, where plants suffered an increase by the TiO_2_ and Ag-TiO_2_ SG treatments in the three organs measured, while the four types NMs synthesized through microwaves had a significant response, indicating a greater interaction of these due to the modification of their synthesis routes. Dunnett’s analysis showed that specific treatments could significantly raise or lower the measured parameters; however, said treatments did not show a significant dependence on the concentration of nanomaterial supplied.

Studies of TiO_2_ interaction with plants have mixed effects where the presence of TiO_2_ enhances some species’ growth of multiple organs such as roots or stems, while other cases show detrimental effects on the growth of these organs [21,22,23,24]. Many mechanisms associated with growth inhibition involve root hair blockage by NMs and damage by ROS, reducing plants’ ability to absorb nutrients, causing reduced plant growth, and multiple morphological traits alteration [25,26,27]. An increased secondary metabolite content could be mainly due to reactive oxygen species (ROS), one of the primary mechanisms described for NMs’ plant interaction and stress induction [9]. The excess of these oxidating molecules affects the vegetable cell disrupting the membrane and unbalancing the plant’s cellular development affecting molecules such as proteins, DNA, and lipids [28]. To mitigate the effect caused by the ROS, the plant synthesizes metabolites that can scavenge the molecules reducing the stress factor caused by the cellular damage, thus increasing its antioxidant activity [29,30], as observed in the results. On the other hand, a study of *Phaseolus vulgaris* L. exposed to CeO_2_ on a solid medium revealed a modification of flavonoid content in roots and leaves. However, polyphenol compounds suffered a pronounced increase in leaves compared to roots, implying that NPs’ effect on secondary metabolisms could have different responses depending on plant tissue [31], as seen before.

In the case of the % inhibition of DPPH, it was observed that some treatments at different concentrations caused a significant increase in the antioxidant activity measured in the three organs. Seen in the two-way ANOVA, the synthesis and dopant factors and the interaction of both were significant, indicating that the effect on the oxidant activity was highly related to the dopant type and the employed synthesis. Although some treatments appear to increase IHB% while having no significant augmentation of the phenol content at several concentrations, this effect could be related to the biostimulation of other secondary metabolism antioxidant molecules that are not measured with the techniques used, such as saponins, which are highly present in alfalfa [32].

The chlorophyll content also suffered a decrease, where it can be observed that the microwave synthesis resulted in a significant reduction due to Cu-TiO_2_, which in the SG synthesis was not the cause of the said effect. On the contrary, the Mw-SG synthesis derived in a TiO_2_ treatment did not generate a significant impact compared to that synthesized by SG. Both synthesis methods coincided in a reduction due to Fe-TiO_2_. Results indicating the reduction or augmentation of photosynthetic pigments in plants treated with TiO_2_ have also been determined [33,34,35,36,37].

Plants with long-term exposure to the NMs can adapt to the stress effects generated by the same NMs, which could alter plant response to NPs [9,38]. As shown in this research, several concentrations did not significantly affect the metabolite content modification. However, this does not imply that alfalfa crops can resist the effect at higher exposure times or NPs with different morphological characteristics. Lower and higher concentrations and comparing metabolite content in different development stages can provide insight into NMs’ plant interaction and secondary metabolism modification. The possibility of TiO_2_ translocation into higher organs could mean different adversities caused by the interaction of NMs with molecules and biochemical processes in different organs. Different alterations in each section depended on NMs’ capability to move through plant tissues, as observed with the metabolic assays. NMs with structural differences appeared to have different degrees of metabolic stress at each organ measured [39].

## 4. Materials and Methods

### 4.1. Titanium Dioxide and Doped TiO_2_ Materials Synthesis and Characterization

Titanium isopropoxide 97% (Sigma Aldrich, St. Louis, MO, USA) was dissolved in isopropanol 99% (J.T. Baker, Phillipsburg, NJ, USA). The solution was stirred for 20 min under a nitrogen atmosphere to prevent the oxidation of the titanium precursor. The hydrolysis process was then performed by adding water to the precursor/solvent solution, and this new solution was stirred for 1 h. For the Ag-modified TiO_2_, the precursor AgNO_3_ (J.T. Baker, Phillipsburg, NJ, USA) was used. For the Fe-TiO_2_, the precursor was FeSO_4_∙7H_2_O (J.T. Baker, Phillipsburg, NJ, USA); for the Cu-TiO_2_, the precursor was CuSO_4_∙5H_2_O (J.T. Baker, Phillipsburg, NJ, USA). These compounds were added by dissolving them into the water used for the hydrolysis in a 0.1%w. The obtained product was dried at room temperature and then calcined at 450 °C for 3 h to promote the anatase crystal phase. For this synthesis, the materials were identified as sol–gel (SG) materials. The TiO_2_ samples synthesized with the microwave-assisted sol–gel method were prepared using the sol obtained after hydrolysis. It was transferred into a Teflon vessel and placed on a microwave reaction system (Flexiwave Milestone). The process was carried out at a temperature of 180 °C for 30 min. Once the product was obtained, it was filtered, dried, and calcined at 450 °C for 3 h [20]. For this synthesis, the materials were identified as microwave-assisted sol–gel (Mw-SG) materials.

Morphology and elemental analysis were carried out using a JEOL JSM-6060 LV scanning electron microscope (SEM) operating at a voltage of 15 keV. Elemental analysis was performed by Energy-Dispersive X-ray Spectroscopy (EDS Oxford Inca X-Sight coupled to a MT 1000, Hitachi). The crystallinity was determined by X-ray Diffraction analysis (XRD) using a Bruker D8 advanced diffractometer equipped with a Cu seal tube to generate Cu Kα radiation (λ = 1.5406 Å) with angles of 10 < 2θ < 80° in a pitch of 0.01°; Raman analysis was conducted using a LabRam HR Horiba Scientific with a NdYGa (λ = 532 nm).

### 4.2. Nanoparticle Crystallite Size

The crystallite size for both synthesis methods was calculated using the Scherrer equation shown in Equation (1), where (D) is the diameter of crystallite, (λ) is the X-ray wavelength, (k) is the Scherrer constant, and (β) is the full width at half maximum obtained from the diffraction signals in the XRD pattern and (θ) the peak position.

(1) $$D=\frac{k\lambda }{\beta \;cos\;cos\;\theta \;}$$

The crystallite size was also determined using the Williamson–Hall equation (Equation (2)), which considers the structural stress of the crystallite. The equation represents a straight line where (ε) is the slope that provides the strain of the crystallite.

(2) $$\beta \;cos\;cos\;\theta =\varepsilon \left(4\;sin\;sin\;\theta \;\right)+\frac{k\lambda }{D}$$

### 4.3. Plant Harvest and Growth Parameters

Alfalfa seeds (*Medicago sativa* L.) were purchased from a local distributor: Hortaflor, Mexico. Seeds were placed in seedbeds using peat moss substrate (Jiffy) with a pH of 5.8, electrical conductivity of 0.4 mS/cm, a moisture fraction of 15%, and particle size of <10 mm inside a plasticized greenhouse of 68 × 49 × 156 cm in length, breadth, and height, respectively. Three replicates with a population of six crops (one crop per container) were maintained during development for the experiment. Sprouts were kept in seedbeds (2.5 × 2.5 × 6.5 cm) for 15 days before being transferred to plastic containers of 500 mL (11.6 cm in height, and 9.5 cm and 6.8 cm in the top and bottom diameter, respectively) using a peat moss substrate. Sprouts were treated by direct soil irrigation with 5 mL solutions of 50, 100, and 500 ppm of TiO_2_ and M-TiO_2_ (M = Ag, Cu, Fe) with no nutritive solution. When transferred to the 500 mL containers, each plant was irrigated with 50 mL of their respective solution of NMs until completing 80 days of treatment.

**Figure 9:**
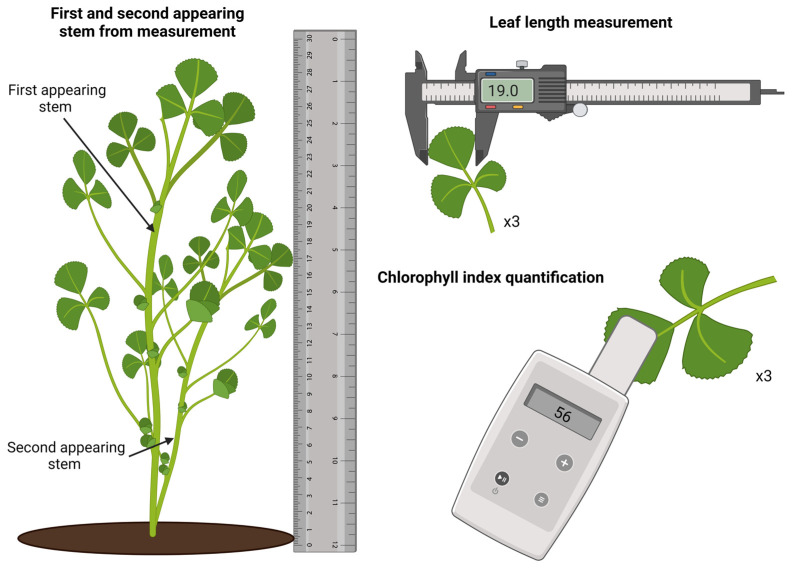
Measurement tools and proceedings for quantifying stem length, leaf length, and chlorophyll index.

Plants were randomly selected for morphological analysis; first and second-appearing stem lengths were measured using a ruler. For leaf length, 3 of the visually biggest trifoliate were chosen in each plant, and their length was measured using a digital caliper, as seen in Figure 9. After harvest, the samples were divided into leaves, stems, and roots immersed in liquid nitrogen to prevent any chemical structural change for future tests. Then, the samples were milled and kept under refrigeration at −20 °C for further metabolomics quantification assays. The greenhouse temperature was recorded using a hygrometer (YASSUN), obtaining temperature and humidity values at midday. Climatic data were taken from the geo-electromagnetic center of the National Autonomous University of Mexico, Juriquilla campus (longitude: 100°26′48.81″ W, latitude: 20°42′14.87″ N) [40] at noon each day. The atmospheric data obtained during the growth period can be visualized in Table S17, present in the supplementary data.

### 4.4. Total Phenol and Flavonoid Quantification

For extract elaboration, 1 g of the fresh frozen sample was weighed and placed in falcon tubes with 10 mL of methanol. The extract was then agitated for 24 h in complete darkness. The extract was then separated and used for total phenol and flavonoid quantification. Total phenolic content was determined according to the Folin–Ciocalteu spectrophotometric method [41] modified for a 96-well microplate. Total phenol content results were expressed as equivalent mg of gallic acid (eq. mg) per gram of fresh sample. Total flavonoid content was expressed as equivalent mg of rutin per gram of fresh sample and was determined by the 2-aminoethyl-diphenyl borate reagent method [42].

#### 4.4.1. Antioxidant Activity

The extracts’ antioxidant activity was evaluated with the 2,2-diphenyl-1-picrylhydrazyl (DPPH) radical method [43], and the results were expressed as the percentage of DPPH discoloration (% radical inhibition) named as well as percentage inhibition (IHB), which was calculated with Equation (3).

(3) $$IHB\%=\left(\frac{A_{DPPH}-A_{S}}{A_{DPPH}}\right)\times 100$$

where (A_S_) is the absorbance of the solution containing the sample, and (A_DPPH_) is the DPPH solution’s absorbance. All the spectrophotometric measurements were obtained in a Thermo Scientific Multiskan Go spectrophotometer.

#### 4.4.2. Chlorophyll Index

The chlorophyll index was quantified using a SPAD 502 Plus Chlorophyll Meter from Minolta Co., Ltd. SPAD values were determined for plants in each treatment group [44,45] taking three readings per plant for an average amount per plant.

### 4.5. Data Analysis

Statistical analysis was performed using the software GraphPad Prism 8 (8.0.2, Dotmatics, San Diego, CA, USA). An ANOVA test was used for morphological and total phenol and flavonoid analysis, and a significant statistical difference was determined using a Dunnett and a Tukey pairwise comparison. The data significance value was *p* ≤ 0.05 in all the analyses.

## 5. Conclusions

The presence of TiO_2_ NMs through constant soil irrigation caused a distressing effect on alfalfa crops, resulting in lower growth rate, alterations in chlorophyll index micronutrient uptake, and increased metabolic profiles. Synthesis pathway modification and different doping elements led to changes in NM characteristics, leading to different metabolomic and morphological alteration levels as proven by the two-way ANOVA analysis. The results suggest that the stress level is influenced by the physicochemical properties of NMs obtained through their synthesis pathway, so TiO_2_-alfalfa interactions with different contents of dopants, exposure, and concentration times are needed to understand the interaction mechanisms and improve TiO_2_ usage as an effective material for crop improvement.

Apart from the distressing effects caused by TiO_2_, NM’s interaction with alfalfa in its multiple presentations caused an outcome in which the secondary metabolite content of total phenols was increased in several treatments, indicating that nanomaterials cause a disruptive interaction with alfalfa crops. The statistical analysis showed that factors such as the dopant, the synthesis method, and the interaction of both significantly influenced parameters such as the chlorophyll index, stem size, % inhibition, and total phenol content (root). Parameters such as the chlorophyll index and stem size showed differences in the types of nanomaterials, generating a decrease in said parameters. However, a significant influence of NMs synthesized by Mw-SG on the total content of phenols and % of inhibition was verified, indicating a modification in the characteristics of the NMs due to the synthesis method, increasing the interaction of said NMs with alfalfa and increasing said parameters.

Further assays for determining NM uptake by roots and translocation are required to address safety concerns related to NMs’ fate through the food chain. Additionally, a deeper understanding of the dose–response models (lineal, threshold, and hormesis) involved in the NP-alfalfa interaction may help understand other toxicological behavior of TiO_2_ and other photocatalytic nanomaterials.

## Figures and Tables

**Table 1:** Crystallite size by Scherrer and Williamson-Hall method and degree of crystallinity for the SG- and Mw-SG-synthesized materials.

Material	Scherrer (nm)		Williamson-Hall (nm)	
	SG	Mw-SG	SG	Mw-SG
TiO_2_	8.65	8.04	19.80	12.49
Ag-TiO_2_	9.62	7.42	14.00	12.27
Fe-TiO_2_	9.28	6.81	18.73	11.45
Cu-TiO_2_	8.75	7.69	16.31	12.27

**Table 2:** Total phenol content in plants treated with SG NMs.

	Leaves			Stem			Root		
	Gallic Acid ^1^ (mg/g)	SD (±)	%	Gallic Acid ^1^ (mg/g)	SD (±)	%	Gallic Acid ^1^ (mg/g)	SD (±)	%
**50 ppm**									
Control	49.21 ^A^	6.82	-	25.40 ^A^	6.73	-	21.52 ^A^	4.10	-
TiO_2_	58.21 ^A^	6.26	18.28	35.13 ^A^	2.46	38.30	36.78 ^B^	2.22	70.91
Ag-TiO_2_	64.75 ^A^	6.31	31.57	29.9 ^A^	2.78	17.78	26.39 ^A^	4.70	22.63
Fe-TiO_2_	62.00 ^A^	6.56	25.99	31.42 ^A^	3.37	23.70	25.94 ^A^	1.58	20.53
Cu-TiO_2_	59.18 ^A^	7.59	20.26	35.90 ^A^	6.40	41.33	24.91 ^A^	1.29	15.75
**100 ppm**									
TiO_2_	84.08 ^B^	5.80	70.85	49.01 ^B^	2.83	92.95	34.65 ^B^	8.12	61.01
Ag-TiO_2_	59.36 ^A^	5.50	20.62	43.72 ^B^	7.87	72.12	34.05 ^B^	6.37	58.22
Fe-TiO_2_	49.99 ^A^	1.87	1.58	34.77 ^A^	6.24	36.88	27.43 ^A^	1.69	27.46
Cu-TiO_2_	63.20 ^A^	9.24	28.42	42.98 ^B^	5.72	69.21	33.21 ^B^	3.72	54.32
**500 ppm**									
TiO_2_	67.03 ^B^	11.27	36.21	49.86 ^B^	9.08	96.29	32.47 ^B^	2.73	50.88
Ag-TiO_2_	52.26 ^A^	4.49	6.19	37.72 ^A^	3.95	48.50	24.65 ^A^	5.91	14.54
Fe-TiO_2_	59.02 ^A^	1.16	19.93	37.59 ^A^	5.72	47.99	25.14 ^A^	3.14	16.82
Cu-TiO_2_	53.62 ^A^	8.24	8.96	38.24 ^A^	3.42	50.55	34.44 ^B^	1.66	60.03

^1^ mg GAE/sample g (mg gallic acid equivalents/sample g). The average represents the value of 3 repetitions. Comparison between means (Dunnett *p* ≤ 0.05). Means with different letters in the same column are statistically different. The percentage columns (%) represent the increase (+) or decrease (−) of the quantified data concerning the control group.

**Table 3:** Total phenol content in plants treated with Mw-SG NMs.

	Leaves			Stem			Root		
	Gallic Acid ^1^ (mg/g)	SD (±)	%	Gallic Acid ^1^ (mg/g)	SD (±)	%	Gallic Acid ^1^ (mg/g)	SD (±)	%
**50 ppm**									
Control	45.72 ^A^	4.01	-	20.48 ^A^	2.38	-	21.71 ^A^	7.76	-
TiO_2_	60.71 ^B^	7.34	32.78	28.27 ^A^	1.92	38.05	29.92 ^A^	5.02	37.81
Ag-TiO_2_	64.08 ^B^	9.87	40.16	24.57 ^A^	2.57	19.97	22.28 ^A^	1.14	2.62
Fe-TiO_2_	59.83 ^B^	3.45	30.86	30.57 ^B^	9.08	49.26	18.09 ^A^	3.87	6.33
Cu-TiO_2_	54.96 ^A^	13.89	20.20	32.46 ^B^	5.85	58.49	29.08 ^A^	3.54	33.94
**100 ppm**									
TiO_2_	67.55 ^B^	5.11	47.74	38.26 ^B^	7.02	86.84	21.80 ^A^	5.39	0.41
Ag-TiO_2_	58.83 ^B^	7.19	28.68	28.09 ^A^	3.14	37.16	27.28 ^A^	4.90	25.65
Fe-TiO_2_	65.81 ^B^	4.58	43.95	34.31 ^B^	6.51	67.53	22.32 ^A^	6.18	2.80
Cu-TiO_2_	72.76 ^B^	5.93	59.14	28.68 ^A^	7.47	40.03	37.00 ^B^	8.77	70.42
**500 ppm**									
TiO_2_	69.45 ^B^	3.10	51.90	26.99 ^A^	1.71	31.78	25.82 ^A^	2.08	18.93
Ag-TiO_2_	58.31 ^B^	4.88	27.53	30.01 ^B^	3.98	46.53	25.64 ^A^	4.60	18.10
Fe-TiO_2_	73.85 ^B^	8.84	61.53	35.12 ^B^	3.16	71.48	29.94 ^A^	6.61	37.89
Cu-TiO_2_	65.67 ^B^	5.88	43.63	46.09 ^B^	8.34	125.04	37.77 ^B^	6.44	73.97

^1^ mg GAE/sample g (mg gallic acid equivalents/sample g). The average represents the value of 3 repetitions. Comparison between means (Dunnett *p* ≤ 0.05). Means with different letters in the same column are statistically different. The percentage columns (%) represent the increase (+) or decrease (−) of the quantified data concerning the control group.

**Table 4:** DPPH inhibition (IHB) % in plants treated with SG NMs.

	Leaves			Stem			Root		
	IHB ^1^ %	SD (±)	%	IHB ^1^ %	SD (±)	%	IHB ^1^ %	SD (±)	%
**50 ppm**									
**Control**	30.66 ^A^	4.08	-	25.65 ^A^	2.19		12.63 ^A^	0.92	-
**TiO_2_**	43.47 ^B^	1.54	41.64	39.28 ^B^	2.02	53.31	19.85 ^B^	3.33	57.16
**Ag-TiO_2_**	43.88 ^A^	2.69	42.97	41.15 ^B^	4.10	60.61	17.41 ^A^	3.19	37.84
**Fe-TiO_2_**	39.86 ^A^	4.27	29.87	29.65 ^A^	1.04	15.72	23.97 ^B^	1.58	89.78
**Cu-TiO_2_**	38.19 ^A^	1.97	24.43	33.68 ^A^	4.17	31.45	17.75 ^A^	5.32	40.53
**100 ppm**									
**TiO_2_**	46.28 ^B^	11.44	50.79	43.76 ^B^	1.26	70.80	22.60 ^B^	0.45	78.93
**Ag-TiO_2_**	40.47 ^A^	4.34	31.86	32.07 ^A^	2.67	25.17	21.13 ^B^	2.36	67.30
**Fe-TiO_2_**	35.32 ^A^	0.54	15.08	28.01 ^A^	1.50	9.32	25.78 ^B^	1.37	104.11
**Cu-TiO_2_**	37.93 ^A^	1.70	23.59	30.15 ^A^	5.69	17.68	15.73 ^A^	2.56	24.54
**500 ppm**									
**TiO_2_**	40.80 ^A^	6.19	32.94	40.41 ^B^	9.60	57.72	20.84 ^B^	2.14	65.00
**Ag-TiO_2_**	41.06 ^A^	6.27	33.79	32.50 ^A^	6.51	26.85	21.20 ^B^	4.04	67.85
**Fe-TiO_2_**	39.10 ^A^	1.52	27.40	32.21 ^A^	2.59	25.72	24.54 ^B^	2.99	94.29
**Cu-TiO_2_**	34.98 ^A^	4.58	13.97	28.33 ^A^	2.42	10.57	21.38 ^B^	2.53	69.27

^1^ DPPH radical inhibition percentage. The average represents the value of 3 repetitions. Comparison between means (Dunnett *p* ≤ 0.05). Means with different letters in the same column are statistically different. The percentage columns (%) represent the increase (+) or decrease (−) of the quantified data concerning the control group.

**Table 5:** DPPH inhibition (IHB)% in plants treated with SG-Mw NMs.

	Leaves			Stem			Root		
	IHB ^1^ %	SD (±)	%	IHB ^1^ %	SD (±)	%	IHB ^1^ %	SD (±)	%
**50 ppm**									
Control	33.93 ^A^	1.78	-	23.24 ^A^	2.58	-	20.40 ^A^	1.71	-
**TiO_2_**	55.88 ^B^	1.80	64.69	36.94 ^B^	1.39	58.95	45.47 ^B^	6.22	122.89
Ag**-TiO_2_**	49.05 ^B^	4.24	44.55	42.09 ^B^	1.46	81.11	26.20 ^A^	2.72	28.43
Fe**-TiO_2_**	44.55 ^B^	1.63	31.29	42.80 ^B^	1.53	84.16	33.80 ^B^	1.36	65.68
Cu**-TiO_2_**	55.91 ^B^	3.43	64.77	42.75 ^B^	4.54	83.95	38.80 ^B^	1.68	90.19
**100 ppm**									
**TiO_2_**	47.49 ^B^	2.96	40.84	37.36 ^B^	1.29	60.76	44.06 ^B^	3.86	115.98
Ag**-TiO_2_**	54.14 ^B^	4.31	59.56	44.35 ^B^	4.08	90.83	39.14 ^B^	2.40	91.86
Fe**-TiO_2_**	43.78 ^B^	2.87	29.03	41.83 ^B^	2.20	79.99	45.12 ^B^	1.98	121.13
Cu**-TiO_2_**	46.74 ^B^	5.51	37.75	31.96 ^B^	2.43	37.55	35.48 ^B^	7.06	73.92
**500 ppm**									
**TiO_2_**	46.67 ^B^	0.91	37.54	34.05 ^B^	1.36	46.51	42.52 ^B^	5.22	108.43
Ag**-TiO_2_**	47.65 ^B^	2.19	40.42	42.17 ^B^	1.41	81.45	38.17 ^B^	3.79	87.10
Fe**-TiO_2_**	46.55 ^B^	3.56	37.20	42.07 ^B^	2.65	81.04	37.28 ^B^	5.50	83.82
Cu**-TiO_2_**	53.19 ^B^	2.64	56.76	47.25 ^B^	2.11	103.31	37.66 ^B^	4.32	84.60

^1^ DPPH radical inhibition percentage. The average represents the value of 3 repetitions. Comparison between means (Dunnett *p* ≤ 0.05). Means with different letters in the same column are statistically different. The percentage columns (%) represent the increase (+) or decrease (−) of the quantified data concerning the control group.

**Table 6:** Two-way ANOVA test for the first appearing stem size.

Factors	ANOVA	SS	DF	MS	F	*p* Value
**Synthesis–Dopant** **(Control)**	Synthesis:dopant	0.3898	4	0.09745	2.980	0.0191 *
	Synthesis	0.1639	1	0.1639	5.012	0.0257 *
	Dopant	1.736	4	0.4341	13.28	0.0001 ***
	Residual	12.82	392	0.03270		
**Synthesis–Dopant** **(No control)**	Synthesis:dopant	0.3703	3	0.1234	3.648	0.0129 *
	Synthesis	0.2614	1	0.2614	7.726	0.0057 **
	Dopant	0.2686	3	0.08955	2.647	0.0489 *
	Residual	12.11	358	0.03383		

* = significant at *p* ≤ 0.05, ** = significant at *p* ≤ 0.01, *** = significant at *p* ≤ 0.001

**Table 7:** Two-way ANOVA test for the total gallic acid content in leaves.

Attribute	ANOVA	SS	DF	MS	F	*p* Value
**Synthesis–Dopant** **(Control)**	Synthesis:dopant	0.2995	4	0.07489	2.190	0.0750 ^ns^
	Synthesis	0.3779	1	0.3779	11.05	0.0012 **
	Dopant	1.167	4	0.2917	8.529	0.0001 ***
	Residual	3.659	107	0.03419		
**Attribute**	**ANOVA table**	**SS**	**DF**	**MS**	**F**	***p*** **value**
**Synthesis–Dopant** **(No control)**	Synthesis:dopant	0.2487	3	0.08289	2.314	0.0805 ^ns^
	Synthesis	0.6614	1	0.6614	18.47	0.0001 ***
	Dopante	0.3924	3	0.1308	3.652	0.0151 *
	Residual	3.582	100	0.03582		

ns = not significant; * = significant at *p* ≤ 0.05, ** = significant at *p* ≤ 0.01, *** = significant at *p* ≤ 0.001

**Table 8:** Two-way ANOVA test for the inhibition % of DPPH in leaves.

Attribute	ANOVA Table	SS	DF	MS	F	*p* Value
**Synthesis–Dopant** **(Control)**	Synthesis:dopant	0.3042	4	0.07605	4.092	0.0040 **
	Synthesis	0.2926	1	0.2926	15.75	0.0001 ***
	Dopant	1.422	4	0.3555	19.13	<0.0001 ****
	Residual	1.970	106	0.01858		
Attribute	**ANOVA table**	**SS**	**DF**	**MS**	**F**	***p*** **value**
**Synthesis–Dopant** **(No control)**	Synthesis:dopant	0.2642	3	0.08805	4.539	0.0050 **
	Synthesis	0.5076	1	0.5076	26.17	0.0001 ***
	Dopant	0.3641	3	0.1214	6.256	0.0006 ***
	Residual	1.921	99	0.01940		

** = significant at *p* ≤ 0.01, *** = significant at *p* ≤ 0.001, **** = significant at *p* ≤ 0.0001.

**Table 9:** Two-way ANOVA test for the chlorophyll index.

Attribute	ANOVA Table	SS	DF	MS	F	*p* Value
**Synthesis–Dopant (Control)**	Synthesis:dopant	1.023	4	0.2558	5.729	0.0002 *
	Synthesis	0.1865	1	0.1865	4.177	0.0416 *
	Dopant	1.521	4	0.3801	8.514	<0.0001 *
	Residual	17.90	401	0.04465		
Attribute	**ANOVA table**	**SS**	**DF**	**MS**	**F**	***p*** **value**
**Synthesis–Dopant (No control)**	Synthesis:dopant	0.9994	3	0.3331	7.326	<0.0001 *
	Synthesis	0.3385	1	0.3385	7.445	0.0067 *
	Dopant	0.6999	3	0.2333	5.131	0.0017 *
	Residual	16.96	373	0.04547		

* = significant at *p* ≤ 0.05

## Data Availability

Data can be requested from the corresponding author (K.E.) if needed.

## Supplementary Materials

The following supporting information can be downloaded at: https://www.mdpi.com/article/10.3390/plants12030659/s1, Table S1, Total flavonoid content in plants treated with SG NMs; Table S2, Total flavonoid content in plants treated with Mw-SG NMs; Table S3, Tukey multiple comparisons for the first appearing stem size; Table S4, Two-way ANOVA test for the second appearing stem length; Table S5, Two-way ANOVA test for the leaf length; Table S6, Tukey multiple comparisons for the total gallic acid content in leaves; Table S7, Two-way ANOVA test for the total gallic acid content in the stem; Table S8, Tukey multiple comparisons for the total gallic acid content in the stem; Table S9, Two-way ANOVA test for the total gallic acid content in roots; Table S10, Tukey multiple comparisons for the total gallic acid content in roots; Table S11, Tukey multiple comparisons for the inhibition % of DPPH in leaves; Table S12, Two-way ANOVA test for the inhibition % of DPPH in the stem; Table S13, Tukey multiple comparisons for the inhibition % of DPPH in the stem; Table S14, Two-way ANOVA test for the inhibition % of DPPH in roots; Table S15, Tukey multiple comparisons for the inhibition % of DPPH in roots; Table S16, Tukey multiple comparisons for chlorophyll index; Table S17, Atmospheric conditions during plant development.
