# Supplementary material for: Medicago sativa L. Plant Response against Possible Eustressors (Fe, Ag, Cu)-TiO2: Evaluation of Physiological Parameters, Total Phenol Content, and Flavonoid Quantification
Source: Plants (Basel). 2023 Feb 2;12(3):659. doi: 10.3390/plants12030659 (PMC9920219; doi:10.3390/plants12030659)
Supplement: Supplementary file 1 [file plants-12-00659-s001.zip › plants-2008841-SI.pdf]

Table S1. Total flavonoid content in plants treated with SG NMs.

	Leaves			Stem			Root		
	**Rutin[1] (mg/g)**	**SD (±)**	**%**	**Rutin[1] (mg/g)**	**SD (±)**	**%**	**Rutin[1] (mg/g)**	**SD (±)**	**%**
**50 ppm**									
**Control**	305.09^{A}	30.61	-	119.06^{A}	19.5	-	11.28^{A}	0.94	-
**TiO_2**	313.27^{A}	90.92	2.68	128.35^{A}	11.91	7.80	16.06^{A}	0.82	42.37
**Ag-TiO_2**	426.8^{A}	53.7	39.89	125.58^{A}	12.23	5.28	31.36^{B}	2.04	178.01
**Fe-TiO_2**	424.2^{A}	49.75	39.04	160.25^{A}	22.78	34.59	20.16^{B}	0.85	78.74
**Cu-TiO_2**	327.5^{A}	26.58	7.35	139.97^{A}	22.12	17.56	15.42^{A}	3.17	36.70
**100 ppm**									
**Control**	305.09^{A}	30.61	-	119.06^{A}	19.5	-	11.28^{A}	0.94	-
**TiO_2**	355.37^{A}	105.50	16.47	174.9^{A}	48.0	46.20	21.04^{B}	0.52	86.52
**Ag-TiO_2**	512.8^{A}	115.55	68.09	165.87^{A}	10.14	39.31	21.02^{B}	4.25	86.40
**Fe-TiO_2**	384.5^{A}	16.06	26.02	142.6^{A}	31.59	19.77	21.59^{B}	2.24	91.40
**Cu-TiO_2**	397.4^{A}	188.11	30.26	134.96^{A}	10.11	13.35	18.04^{A}	4.64	59.91
**500 ppm**									
**Control**	305.09^{A}	30.61	-	119.06^{A}	19.5	-	11.28^{A}	0.94	-
**TiO_2**	345.83^{A}	78.13	13.35	146.46^{A}	13.67	23.01	21.09^{B}	3.39	86.86
**Ag-TiO_2**	330.0^{A}	69.22	8.18	143.3^{A}	37.4	20.35	11.66^{A}	2.23	3.36
**Fe-TiO_2**	393.5^{A}	55.83	28.99	165.44^{A}	27.8	38.95	19.78^{B}	2.17	75.35
**Cu-TiO_2**	404.5^{A}	32.92	32.58	167.60^{A}	28.40	40.76	19.90^{B}	2.19	76.41

[1] mg RE/sample g (Rutine Equivalents/sample g). The average represents the value of 3 repetitions. Comparison between means (Dunnett $p \leq 0.05$). Means with different letters in the same column are statistically different. The percentage columns (%) represent the increase (+) or decrease (-) of the quantified data concerning the control group.

Table S2. Total flavonoid content in plants treated with Mw-SG NMs.

	Leaves			Stem			Root		
	**Rutin[1] (mg/g)**	**SD (±)**	**%**	**Rutin[1] (mg/g)**	**SD (±)**	**%**	**Rutin[1] (mg/g)**	**SD (±)**	**%**
				**50 ppm**					
**Control**	396.54^{A}	57.90	-	132.03^{A}	10.09	-	19.55^{A}	1.93	-
**TiO_2**	540.43^{A}	113.01	36.28	146.83^{A}	8.30	11.21	23.84^{A}	2.74	21.94
**Ag-TiO_2**	400.75^{A}	64.27	1.06	141.14^{A}	12.71	6.89	24.69^{A}	3.91	26.29
**Fe-TiO_2**	529.41^{A}	59.66	33.50	165.93^{A}	10.54	25.67	21.44^{A}	3.43	9.66
**Cu-TiO_2**	540.57^{A}	89.75	36.32	142.64^{A}	34.04	8.04	24.01^{A}	2.85	22.81
				**100 ppm**					
**Control**	396.54^{A}	57.90	-	132.03^{A}	10.09	-	19.55^{A}	1.93	-
**TiO_2**	401.23B^{A}	24.29	21.35	162.61^{A}	9.59	23.16	24.20^{A}	4.15	23.78
**Ag-TiO_2**	504.95^{A}	93.32	27.33	176.70^{B}	19.17	33.83	25.83^{A}	4.16	32.12
**Fe-TiO_2**	574.86^{B}	82.32	44.96	227.5^{B}	40.93	72.30	26.09^{A}	2.11	33.45
**Cu-TiO_2**	534.76^{A}	58.56	34.85	164.16^{A}	19.06	24.33	24.4^{A}	4.82	24.80
				**500 ppm**					
**Control**	396.54^{A}	57.90	-	132.03^{A}	10.09	-	19.55^{A}	1.93	-
**TiO_2**	408.19^{A}	42.08	2.93	136.10^{A}	11.50	3.08	25.48^{A}	4.38	30.33
**Ag-TiO_2**	442.14^{A}	24.20	11.49	172.88^{B}	10.87	30.94	21.27^{A}	1.49	8.79
**Fe-TiO_2**	688.59^{B}	185.01	73.65	176.21^{B}	18.55	33.46	22.33^{A}	1.59	14.21
**Cu-TiO_2**	504.01^{A}	42.06	27.10	174.86^{B}	16.76	32.44	27.76^{B}	3.07	41.99

[1]mg RE/sample g (Rutine Equivalents/sample g). The average represents the value of 3 repetitions. Comparison between means ((Dunnett $p \leq 0.05$). Means with different letters in the same column are statistically different. The percentage columns (%) represent the increase (+) or decrease (-) of the quantified data concerning the control group.

Table S3. Tukey multiple comparisons for the first appearing stem size.

Tukey's multiple comparisons tests	Mean Diff.	Summary	Adjusted P Value
	**SG NMs**		
Control vs. Ti	0.2975	****	<0.0001
Control vs. Ag	0.236	****	<0.0001
Control vs. Cu	0.1629	*	0.0173
Control vs. Fe	0.2535	****	<0.0001
Ti vs. Ag	-0.0615	ns	0.6689
Ti vs. Cu	-0.1346	*	0.0314
Ti vs. Fe	-0.04408	ns	0.8394
Ag vs. Cu	-0.07313	ns	0.4328
Ag vs. Fe	0.01743	ns	0.9917
Cu vs. Fe	0.09056	ns	0.1445
	**SG-MW NMs**		
Control vs. Ti	0.1448	*	0.0283
Control vs. Ag	0.1549	*	0.0159
Control vs. Cu	0.1895	**	0.0013
Control vs. Fe	0.2399	****	<0.0001
Ti vs. Ag	0.01009	ns	0.9985
Ti vs. Cu	0.04466	ns	0.7054
Ti vs. Fe	0.09511	*	0.0493
Ag vs. Cu	0.03457	ns	0.8644
Ag vs. Fe	0.08502	ns	0.1093
Cu vs. Fe	0.05045	ns	0.596

ns = not significant; * = significant at $p \le 0.05$, ** = significant at $p \le 0.01$, *** = significant at $p \le 0.001$, **** = significant at $p \le 0.0001$.

Table S4. Two-way ANOVA test for the second appearing stem length.

Attribute	ANOVA	SS	DF	MS	F	P value
**Synthesis-Dopant (Control)**	Synthesis:dopant	0.6259	4	0.1565	3.037	0.0175*
	Synthesis	2.306	1	2.306	44.75	0.0001***
	Dopant	2.698	4	0.6744	13.09	0.0001***
	Residual	18.96	368	0.05153		
**Attribute**	**ANOVA table**	**SS**	**DF**	**MS**	**F**	**P value**
**Synthesis-Dopant (No control)**	Synthesis:dopant	0.2785	3	0.09284	1.771	0.1524^{ns}
	Synthesis	3.642	1	3.642	69.48	0.0001***
	Dopante	0.3451	3	0.1150	2.195	0.0885^{ns}
	Residual	17.56	335	0.05241		

ns = not significant; * = significant at $p \leq 0.05$, ** = significant at $p \leq 0.01$, *** = significant at $p \leq 0.001$, **** = significant at $p \leq 0.0001$.

Table S5. Two-way ANOVA test for the leaf length.

Tukey's multiple comparisons tests	Mean Diff.	Summary	Adjusted P Value
	**SG NMs**		
Control vs. Ti	0.424	****	<0.0001
Control vs. Ag	0.3339	****	<0.0001
Control vs. Cu	0.3158	****	<0.0001
Control vs. Fe	0.4288	****	<0.0001
Ti vs. Ag	-0.09014	ns	0.5305
Ti vs. Cu	-0.1082	ns	0.3608
Ti vs. Fe	0.004854	ns	>0.9999
Ag vs. Cu	-0.01811	ns	0.9975
Ag vs. Fe	0.09499	ns	0.3285
Cu vs. Fe	0.1131	ns	0.1884
	**SG-MW NMs**		
Control vs. Ti	0.117	ns	0.3353
Control vs. Ag	0.185	*	0.0272
Control vs. Cu	0.1473	ns	0.1285
Control vs. Fe	0.2053	**	0.0089
Ti vs. Ag	0.06802	ns	0.574
Ti vs. Cu	0.03031	ns	0.9639
Ti vs. Fe	0.08824	ns	0.2871
Ag vs. Cu	-0.03771	ns	0.9223
Ag vs. Fe	0.02022	ns	0.9915
Cu vs. Fe	0.05793	ns	0.6948

ns = not significant; * = significant at $p \leq 0.05$, ** = significant at $p \leq 0.01$, *** = significant at $p \leq 0.001$, **** = significant at $p \leq 0.0001$.

Table S6. Tukey multiple comparisons for the total gallic acid content in leaves.

Tukey's multiple comparisons tests	Mean Diff.	Summary	Adjusted P Value
	SG NMs		
Control vs. Ti	-0.4179	**	0.0085
Control vs. Ag	-0.1947	ns	0.5139
Control vs. Cu	-0.1922	ns	0.5268
Control vs. Fe	-0.1584	ns	0.7008
Ti vs. Ag	0.2232	ns	0.0853
Ti vs. Cu	0.2256	ns	0.0797
Ti vs. Fe	0.2594	*	0.0291
Ag vs. Cu	0.002487	ns	>0.9999
Ag vs. Fe	0.03626	ns	0.9936
Cu vs. Fe	0.03377	ns	0.9952
	Mw-SG NMs		
Control vs. Ti	-0.4414	****	<0.0001
Control vs. Ag	-0.3213	**	0.0033
Control vs. Cu	-0.41	****	<0.0001
Control vs. Fe	-0.4545	****	<0.0001
Ti vs. Ag	0.1202	ns	0.2981
Ti vs. Cu	0.03143	ns	0.9862
Ti vs. Fe	-0.0131	ns	0.9995
Ag vs. Cu	-0.08872	ns	0.6038
Ag vs. Fe	-0.1333	ns	0.2023
Cu vs. Fe	-0.04453	ns	0.9509

ns = not significant; * = significant at $p \leq 0.05$, ** = significant at $p \leq 0.01$, *** = significant at $p \leq 0.001$, **** = significant at $p \leq 0.0001$.

Table S7. Two-way ANOVA test for the total gallic acid content in the stem.

Attribute	ANOVA	SS	DF	MS	F	P value
**Synthesis-Dopant (Control)**	Synthesis:dopant	0.6121	4	0.1530	1.189	0.3197^{ns}
	Synthesis	0.09561	1	0.09561	0.7431	0.3906^{ns}
	Dopant	2.678	4	0.6695	5.204	0.0007***
	Residual	13.77	107	0.1287		
**Attribute**	**ANOVA table**	**SS**	**DF**	**MS**	**F**	**P value**
**Synthesis-Dopant (No control)**	Synthesis:dopant	0.5992	3	0.1997	1.473	0.2264^{ns}
	Synthesis	0.1673	1	0.1673	1.234	0.2693^{ns}
	Dopante	0.6971	3	0.2324	1.714	0.1690^{ns}
	Residual	13.56	100	0.1356		

ns = not significant; * = significant at $p \leq 0.05$, ** = significant at $p \leq 0.01$, *** = significant at $p \leq 0.001$, **** = significant at $p \leq 0.0001$.

Table S8. Tukey multiple comparisons for the total gallic acid content in the stem.

Tukey's multiple comparisons tests	Mean Diff.	Summary	Adjusted P Value
**SG NMs**			
Control vs. Ti	-0.5443	ns	0.1608
Control vs. Ag	-0.4613	ns	0.3085
Control vs. Cu	-0.5371	ns	0.171
Control vs. Fe	-0.3621	ns	0.5556
Ti vs. Ag	0.08297	ns	0.9881
Ti vs. Cu	0.007151	ns	>0.9999
Ti vs. Fe	0.1821	ns	0.8179
Ag vs. Cu	-0.07581	ns	0.9915
Ag vs. Fe	0.09917	ns	0.9768
Cu vs. Fe	0.175	ns	0.8386
**SG-MW NMs**			
Control vs. Ti	-0.5203	*	0.0219
Control vs. Ag	-0.3456	ns	0.2525
Control vs. Cu	-0.7453	***	0.0002
Control vs. Fe	-0.6275	**	0.003
Ti vs. Ag	0.1747	ns	0.5899
Ti vs. Cu	-0.225	ns	0.3332
Ti vs. Fe	-0.1072	ns	0.8975
Ag vs. Cu	-0.3997	**	0.0099
Ag vs. Fe	-0.2819	ns	0.1352
Cu vs. Fe	0.1178	ns	0.8614

ns = not significant; * = significant at p ≤ 0.05, ** = significant at p ≤ 0.01, *** = significant at p ≤ 0.001, **** = significant at p ≤ 0.0001.

Table S9. Two-way ANOVA test for the total gallic acid content in roots.

Attribute	ANOVA	SS	DF	MS	F	P value
**Synthesis-Dopant (Control)**	Synthesis:dopant	1.044	4	0.2611	3.501	0.0100*
	Synthesis	0.2663	1	0.2663	3.570	0.0615ns
	Dopant	2.710	4	0.6776	9.084	0.0001***
	Residual	7.981	107	0.07459		
**Attribute**	**ANOVA table**	**SS**	**DF**	**MS**	**F**	**P value**
**Synthesis-Dopant (No control)**	Synthesis:dopant	1.009	3	0.3362	4.625	0.0045**
	Synthesis	0.4660	1	0.4660	6.409	0.0129*
	Dopante	1.933	3	0.6444	8.864	0.0001***
	Residual	7.270	100	0.07270		

ns = not significant; * = significant at p ≤ 0.05, ** = significant at p ≤ 0.01, *** = significant at p ≤ 0.001, **** = significant at p ≤ 0.0001.

Table S10. Tukey multiple comparisons for the total gallic acid content in roots.

Tukey's multiple comparisons tests	Mean Diff.	Summary	Adjusted P Value
SG NMs			
Control vs. Ti	-0.6085	**	0.0099
Control vs. Ag	-0.3178	ns	0.4108
Control vs. Cu	-0.4339	ns	0.1279
Control vs. Fe	-0.2158	ns	0.7598
Ti vs. Ag	0.2907	ns	0.1669
Ti vs. Cu	0.1746	ns	0.6568
Ti vs. Fe	0.3927	*	0.0236
Ag vs. Cu	-0.1161	ns	0.8956
Ag vs. Fe	0.102	ns	0.9323
Cu vs. Fe	0.2181	ns	0.4418
SG-MW NMs			
Control vs. Ti	-0.1903	ns	0.5789
Control vs. Ag	-0.1545	ns	0.7516
Control vs. Cu	-0.5941	***	0.0001
Control vs. Fe	-0.07989	ns	0.9715
Ti vs. Ag	0.03585	ns	0.9948
Ti vs. Cu	-0.4038	***	0.0002
Ti vs. Fe	0.1104	ns	0.744
Ag vs. Cu	-0.4397	****	<0.0001
Ag vs. Fe	0.07456	ns	0.9243
Cu vs. Fe	0.5143	****	<0.0001

ns = not significant; * = significant at $p \leq 0.05$, ** = significant at $p \leq 0.01$, *** = significant at $p \leq 0.001$, **** = significant at $p \leq 0.0001$.

Table S11. Tukey multiple comparisons for the inhibition % of DPPH in leaves.

Tukey's multiple comparisons tests	Mean Diff.	Summary	Adjusted P Value
	SG NMs		
Control vs. Ti	-0.4192	***	0.0001
Control vs. Ag	-0.355	**	0.0019
Control vs. Cu	-0.2077	ns	0.1576
Control vs. Fe	-0.2423	ns	0.066
Ti vs. Ag	0.0642	ns	0.8684
Ti vs. Cu	0.2114	*	0.0116
Ti vs. Fe	0.1769	ns	0.053
Ag vs. Cu	0.1472	ns	0.1793
Ag vs. Fe	0.1127	ns	0.4377
Cu vs. Fe	-0.03457	ns	0.9832
	SG-MW NMs		
Control vs. Ti	-0.474	****	<0.0001
Control vs. Ag	-0.4818	****	<0.0001
Control vs. Cu	-0.5309	****	<0.0001
Control vs. Fe	-0.3252	****	<0.0001
Ti vs. Ag	-0.007806	ns	0.9998
Ti vs. Cu	-0.05692	ns	0.7205
Ti vs. Fe	0.1488	*	0.0122
Ag vs. Cu	-0.04912	ns	0.816
Ag vs. Fe	0.1566	**	0.0071
Cu vs. Fe	0.2058	***	0.0002

ns = not significant; * = significant at $p \leq 0.05$, ** = significant at $p \leq 0.01$, *** = significant at $p \leq 0.001$, **** = significant at $p \leq 0.0001$.

Table S12. Two-way ANOVA test for the inhibition % of DPPH in the stem.

Attribute	ANOVA	SS	DF	MS	F	P value
**Synthesis-Dopant (Control)**	Synthesis:dopant	1.896	4	0.4740	15.23	0.0001***
	Synthesis	2.267	1	2.267	72.84	0.0001***
	Dopant	2.411	4	0.6026	19.36	0.0001***
	Residual	3.299	106	0.03112		
**Attribute**	**ANOVA table**	**SS**	**DF**	**MS**	**F**	**P value**
**Synthesis-Dopant (No control)**	Synthesis:dopant	1.574	3	0.5248	16.12	0.0001***
	Synthesis	3.933	1	3.933	120.8	0.0001***
	Dopante	0.2951	3	0.09836	3.022	0.0333**
	Residual	3.223	99	0.03255		

ns = not significant; * = significant at $p \leq 0.05$, ** = significant at $p \leq 0.01$, *** = significant at $p \leq 0.001$, **** = significant at $p \leq 0.0001$.

Table S13. Tukey multiple comparisons for the inhibition % of DPPH in the stem.

Tukey's multiple comparisons tests	Mean Diff.	Summary	Adjusted P Value
	SG NMs		
**Control vs. Ti**	-0.5877	****	<0.0001
**Control vs. Ag**	-0.3739	*	0.0162
**Control vs. Cu**	-0.1977	ns	0.4499
**Control vs. Fe**	-0.1679	ns	0.6116
**Ti vs. Ag**	0.2138	ns	0.0997
**Ti vs. Cu**	0.39	***	0.0001
**Ti vs. Fe**	0.4199	****	<0.0001
**Ag vs. Cu**	0.1762	ns	0.2198
**Ag vs. Fe**	0.2061	ns	0.1035
**Cu vs. Fe**	0.02986	ns	0.9964
	SG-MW NMs		
**Control vs. Ti**	-0.5536	****	<0.0001
**Control vs. Ag**	-0.8442	****	<0.0001
**Control vs. Cu**	-0.7488	****	<0.0001
**Control vs. Fe**	-0.8166	****	<0.0001
**Ti vs. Ag**	-0.2906	****	<0.0001
**Ti vs. Cu**	-0.1952	*	0.0106
**Ti vs. Fe**	-0.263	***	0.0002
**Ag vs. Cu**	0.09533	ns	0.4873
**Ag vs. Fe**	0.02757	ns	0.99
**Cu vs. Fe**	-0.06777	ns	0.7781

ns = not significant; * = significant at $p \leq 0.05$, ** = significant at $p \leq 0.01$, *** = significant at $p \leq 0.001$, **** = significant at $p \leq 0.0001$.

Table S14. Two-way ANOVA test for the inhibition % of DPPH in roots.

Attribute	ANOVA	SS	DF	MS	F	P value
**Synthesis-Dopant (Control)**	Synthesis:dopant	1.229	4	0.3073	4.623	0.0018**
	Synthesis	0.7015	1	0.7015	10.55	0.0016**
	Dopant	6.473	4	1.618	24.34	0.0001***
	Residual	7.047	106	0.06648		
**Attribute**	**ANOVA table**	**SS**	**DF**	**MS**	**F**	**P value**
**Synthesis-Dopant (No control)**	Synthesis:dopant	1.134	3	0.3781	5.347	0.0019**
	Synthesis	1.225	1	1.225	17.32	0.0001***
	Dopante	2.006	3	0.6688	9.458	0.0001***
	Residual	7.001	99	0.07071		

ns = not significant; * = significant at $p \leq 0.05$, ** = significant at $p \leq 0.01$, *** = significant at $p \leq 0.001$, **** = significant at $p \leq 0.0001$

Table S15. Tukey multiple comparisons for the inhibition % of DPPH in roots.

Tukey's multiple comparisons tests	Mean Diff.	Summary	Adjusted P Value
	SG NMs		
**Control vs. Ti**	-0.6724	**	0.0015
**Control vs. Ag**	-0.576	**	0.0096
**Control vs. Cu**	-0.4471	ns	0.0774
**Control vs. Fe**	-0.9599	****	<0.0001
**Ti vs. Ag**	0.09639	ns	0.9321
**Ti vs. Cu**	0.2253	ns	0.3486
**Ti vs. Fe**	-0.2875	ns	0.1331
**Ag vs. Cu**	0.1289	ns	0.826
**Ag vs. Fe**	-0.3839	*	0.0173
**Cu vs. Fe**	-0.5128	***	0.0005
	SG-MW NMs		
**Control vs. Ti**	-1.157	****	<0.0001
**Control vs. Ag**	-0.6774	****	<0.0001
**Control vs. Cu**	-0.8287	****	<0.0001
**Control vs. Fe**	-0.8983	****	<0.0001
**Ti vs. Ag**	0.4796	****	<0.0001
**Ti vs. Cu**	0.3284	**	0.0021
**Ti vs. Fe**	0.2588	*	0.0264
**Ag vs. Cu**	-0.1512	ns	0.4178
**Ag vs. Fe**	-0.2208	ns	0.0912
**Cu vs. Fe**	-0.0696	ns	0.9271

ns = not significant; * = significant at $p \leq 0.05$, ** = significant at $p \leq 0.01$, *** = significant at $p \leq 0.001$, **** = significant at $p \leq 0.0001$.

Table S16. Tukey multiple comparisons for chlorophyll index.

Tukey's multiple comparisons tests	Mean Diff.	Summary	Adjusted P Value
	SG NMs		
**Control vs. Ti**	0.3131	****	<0.0001
**Control vs. Ag**	0.1596	ns	0.1446
**Control vs. Cu**	0.1283	ns	0.3434
**Control vs. Fe**	0.2202	*	0.0135
**Ti vs. Ag**	-0.1536	**	0.0084
**Ti vs. Cu**	-0.1848	***	0.0007
**Ti vs. Fe**	-0.09292	ns	0.2606
**Ag vs. Cu**	-0.03126	ns	0.9611
**Ag vs. Fe**	0.06064	ns	0.682
**Cu vs. Fe**	0.0919	ns	0.2712
	SG-MW NMs		
**Control vs. Ti**	0.1139	ns	0.2823
**Control vs. Ag**	0.05555	ns	0.8703
**Control vs. Cu**	0.2083	**	0.0031
**Control vs. Fe**	0.2034	**	0.0041
**Ti vs. Ag**	-0.05833	ns	0.6146
**Ti vs. Cu**	0.09438	ns	0.1507
**Ti vs. Fe**	0.08955	ns	0.1889
**Ag vs. Cu**	0.1527	**	0.002
**Ag vs. Fe**	0.1479	**	0.0029
**Cu vs. Fe**	-0.004828	ns	>0.9999

ns = not significant; * = significant at $p \leq 0.05$, ** = significant at $p \leq 0.01$, *** = significant at $p \leq 0.001$, **** = significant at $p \leq 0.0001$.

Table S17. Atmospheric conditions during plant development

**SG NMs**		**may-18**	**jun-18**	**jul-18**	**ago-18**
	Temperature (°C)	27.69	24.69	25.2	24.65
	Humidity (%)	30.22	44.59	41.8	43.5
	Heat index (°C)	26.52	24.124	24.73	24.26
	Solar radiation (W/m^2)	923.9	726.62	839.77	855.73
	UV index	7.42	6.28	7.66	8.08
**Mw-SG NMs**		may-19	jun-19	jul-19	ago-19
	Temperature	26.79	26.18	25.88	26.93
	Humidity	35.23	49.3	41.64	36.51
	Indice de calor	25.58	25.4	25.38	26.27
	Radiación solar	878.6	1014	855.48	724.48
	Indice UV	5.66	7.6	7.49	6.21
